# Evaluation of the emergency medical system in an area following lifting of the mandatory evacuation order after the Fukushima Daiichi Nuclear Power Plant accident: A retrospective cross-sectional observational study

**DOI:** 10.1097/MD.0000000000026466

**Published:** 2021-06-25

**Authors:** Chika Yamamoto, Toyoaki Sawano, Yoshitaka Nishikawa, Akihiko Ozaki, Yuki Shimada, Tomohiro Morita, Tianchen Zhao, Arifumi Hasegawa, Tomoyoshi Oikawa, Masaharu Tsubokura

**Affiliations:** a Department of Emergency, Minamisoma Municipal General Hospital; b Division of Disaster and Radiation Medical Sciences, Fukushima Medical University; c Department of Surgery, Jyoban Hospital of Tokiwa Foundation; d Department of Radiation Health Management, Fukushima Medical University School of Medicine; e Research Center for Community Health, Minamisoma Municipal General Hospital; f Department of Internal Medicine, Soma Central Hospital, Fukushima; g Department of Health Informatics, Kyoto University School of Public Health, Kyoto; h Department of Breast Surgery, Jyoban Hospital of Tokiwa Foundation; i Department of Neurosurgery, Minamisoma Municipal General Hospital, Fukushima; j Medical Governance Research Institute, Tokyo; k Department of Radiation Disaster Medicine, Fukushima Medical University School of Medicine, Fukushima, Japan.

**Keywords:** evacuation zone, Fukushima Nuclear Disaster, radiation disaster

## Abstract

Following the lifting of the evacuation order due to the Fukushima Daiichi Nuclear Power Plant accident, the medical demand and emergency medical system (EMS) in the area where the evacuation orders were lifted have not been well-investigated. This study aimed to evaluate the emergency transportation in such areas and compare the differences with areas that had minimal impact.

Using the local EMS transport records, the characteristics of patients who were transferred by an EMS vehicle in Minamisoma City were collected between July 12, 2016 and July 31, 2018, and were compared between former evacuation zones and outside the evacuation zones in the city.

The number of emergency transports in the study period in Minamisoma City were 325 cases in the area where the evacuation orders were lifted and 4307 cases in the other areas. The total EMS time was significantly longer in the area where the evacuation order was lifted (48 ± 16 minutes) than in the other areas (40 ± 15 minutes) (*P* < .001). In the analysis of each component of EMS times, the transport time, which is the time from departure from the patient's location to arrival at a hospital, was significantly longer in the former evacuation zone than in the other areas (16 ± 9 vs 9 ± 9 minutes, *P* < .001), suggesting that transport time contributed to the longer EMS response times.

In areas where the evacuation orders were lifted, the EMS transport time was significantly longer than that in the area outside the former evacuation zone; correspondingly, the total EMS time significantly increased in the former evacuation zone. A plausible reason for this may be the closure of local medical facilities following the evacuation order after the nuclear accident.

## 1 Introduction

Maintenance of Emergency Medical Service (EMS) systems, such as emergency transport and emergency care, is vital for the public health of local residents.^[1–5]^ EMS time is one of the important public health indicators since a delay in the EMS time will worsen the prognosis of patients with severe illnesses or injuries, such as multiple trauma, acute myocardial infarction, and cerebral infarction.^[6–9]^ Although prolonged EMS times may be attributed to factors, such as lack of medical resources, external factors, such as geographical distance, and internal factors, such as hospital closures, it is of paramount importance to identify the causes of prolonged EMS time with the aim of developing effective interventions, especially within resource-limited rural and remote areas.^[10–12]^

In the wake of emergencies, such as natural and man-made disasters, terrorist attacks, and adverse weather events, EMS may be impacted in various ways.^[2,13–17]^ Previous reports have indicated that even though medical institution functions may be preserved after a mass disaster, emergency transport is directly hampered due to traffic congestion or disruption of roads.^[18–22]^ The medical institutions’ functions themselves may also be impacted by earthquakes, tsunamis etc. Human resources may be limited due to staff evacuation, leading to delays of emergency transport.^[19,23]^ However, these reports have mainly focused on the period immediately after the disaster, with very little information available on the situation of emergency transport during the disaster recovery period.

After the Great East Japan Earthquake and subsequent tsunami in March 2011, followed by the Fukushima Daiichi Nuclear Power Plant (FDNPP) accident, evacuation orders were issued over a wide area of costal district of Fukushima Prefecture (Soma and Futaba District), where the FDNPP is located. In some areas, the evacuation orders have been lifted, and some former residents have returned. In some areas, the evacuation orders have been lifted, and some former residents have returned.^[10]^ Most of the returning residents are the elderly, making resumption of medical systems in the former evacuation areas a priority.^[24,25]^ Health care systems in the affected areas remain heavily affected since many hospitals in the area were forced to close and remain closed even after the evacuation orders were lifted.^[23]^ Notably, the situations of emergency transport in the area where the evacuation order was lifted have been shown to be different from those before the disaster, leading to the delay in EMS time.^[23,24,26,27]^ Thus, in developing effective countermeasures for nuclear disaster recovery, it is important to accumulate evidence on the medical demand and the state of emergency transport in the area where the evacuation orders were lifted.

This study evaluated the characteristics of emergency transport in Odaka Ward in Minamisoma City, where the evacuation orders were lifted in 2016 after the FDNPP accident. The details of emergency transport were also compared between the affected areas and other parts of the city that were minimally impacted. The results of this study will help in understanding the impact of evacuation orders during a disaster on emergency transport.

## 2 Methods

### 2.1 Design and setting

**Figure 1:**
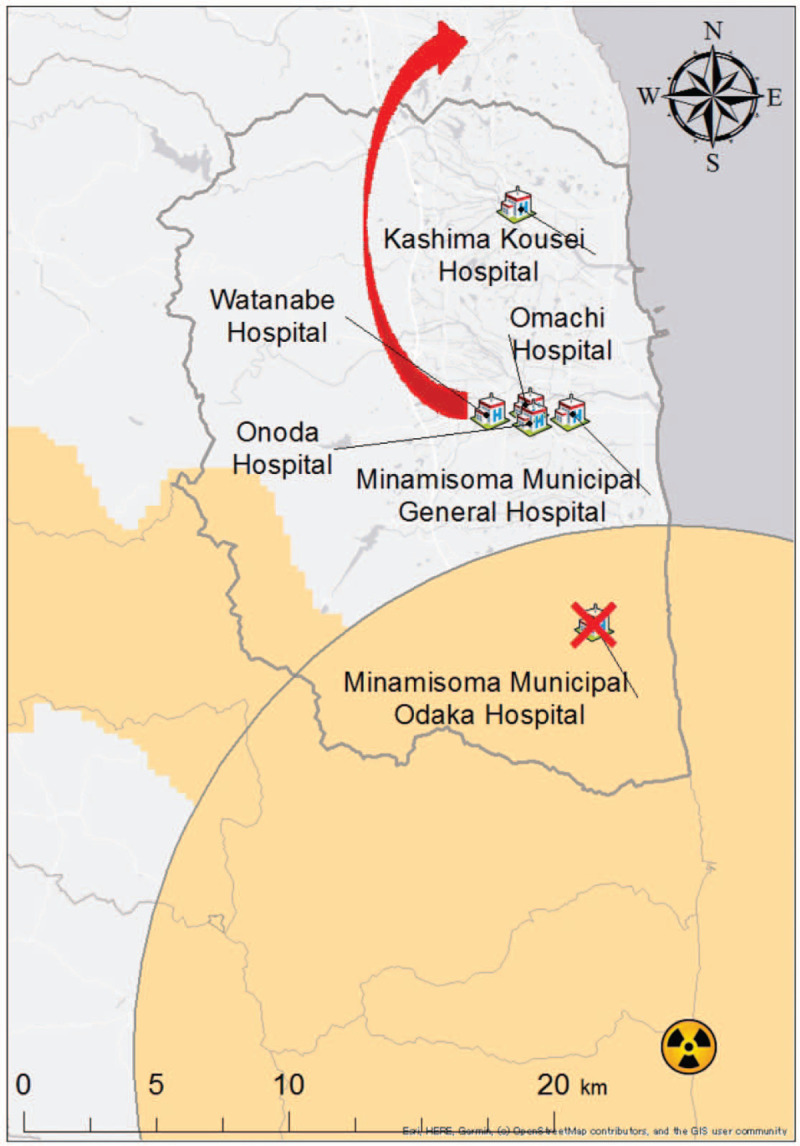
The impact on hospitals with a hospitalization function in Minamisoma City after the evacuation order in the Fukushima Daiichi Nuclear accident (except hospitals for psychiatry patients). The area where the evacuation order was issued after the Fukushima Daiichi Nuclear Power Plant accident is colored orange. There were five hospitals with hospitalization functions in Minamisoma City, Fukushima Prefecture, before the Fukushima Daiichi Nuclear Power Plant accident, except hospitals for psychiatry patients. Two hospitals, Watanabe Hospital and Minamisoma Municipal Odaka Hospital, were forced to suspend their hospitalization function. After continuing with outpatient treatment in Minamisoma City, Watanabe Hospital moved to another municipality in the Soma district, thereby resuming its hospitalization function.

This was a retrospective observational study of patients who were transferred by an EMS vehicle after EMS calls in the area where the evacuation orders were lifted after the FDNPP accident. Minamisoma City is located 11 to 38 km north of the FDNPP and is in the Soma District (Fig. 1).

Following the FDNPP accident, the Japanese government divided Minamisoma City into 4 areas: Evacuation Order Zone, which is within a 20 km radius from the FDNPP; Planned Evacuation Zone for the western part of the city outside the 20 km radius; Emergency Evacuation Preparation Zone for a 20 to 30 km radius from the FDNPP; Non-Evacuation Zone for areas beyond a 30 km radius from the FDNPP. Odaka Ward, the southern part of Minamisoma City, is located within 20 km from the FDNPP.^[28]^

After the evacuation order had been transiently changed to “Evacuation Order Cancellation Preparation Zone," the evacuation orders were lifted on July 12, 2016. As a result, some of the former residents gradually initiated a return to Odaka Ward. Odaka Ward had 12,840 residents on March 11, 2011, before the accident. As of July 31, 2018, 2875 residents lived in the ward, and 1425 (49.5%) of the residents were >65 years. This number accounts for 22% of the original population.^[29]^

Notably, the FDNPP accident drastically affected the distribution of medical institutions in Minamisoma City, including Odaka Ward, because of relocation and closure of hospitals (with hospitalization facility) and clinics (without hospitalization facility). As of August 2018, the number of hospitals, except for hospitals specializing in psychiatry, in the whole of Minamisoma City decreased from 8 to 6, and that of clinics decreased from 39 to 31 compared to that before the accident. Particularly in Odaka Ward, the only hospital with a hospitalization facility before the accident was closed after the accident. This hospital reopened only for outpatient services from April 2014, but the number of clinics also decreased from 7 to 3 after the accident (Fig. 1). The number of hospital beds, except for psychiatry patients in Minamisoma City, was 695 at the time of the disaster, which decreased to 386 as of August 2018.^[30]^

In Japan, local fire departments are generally required to assume EMS, such as transporting patients and seeking hospitals, which accept such patients based on the Fire Service Act. Fire departments are located in each local municipality under the prefectural government and usually have one or more ambulances. Once an EMS call is received, an ambulance with the EMS team departs from the nearest fire department to the patient's location, and the EMS team triages the patient and subsequently seeks a hospital that can accept the patient. After determining a destination hospital, the ambulance with the patient leaves the patient's location to the hospital. In the case where no hospital is able to accept the patient, the EMS team repeatedly contacts hospitals in the local or surrounding areas.

### 2.2 Data collection

The EMS transport records of the Soma Regional Fire Department were used to collect data on EMS transport for patients who were transferred by an EMS vehicle after EMS calls for the period between July 12, 2016 and July 31, 2018. All of the variables in the emergency transport record from the Fire Department of Soma are listed in the Supplemental Digital Material (). The observation period was defined based on the fact July 12, 2016 was the day of lifting of the evacuation orders in Odaka Ward and on the assumption that approximately 2 years after the lifting of evacuation orders would provide useful information around this topic. Patients who were not transported and those who were transferred between hospitals were excluded. Transportations from Minamisoma City were divided into two categories: from areas where the evacuation orders were lifted (referred to as the “former evacuation zone”) and from other unaffected areas (referred to as “outside the evacuation zone”).

For each EMS transport, seven items were extracted: EMS time, time of day of the EMS call (0:00–5:59, 6:00–11:59, 12:00–17:59, 18:00–23:59), sex (male, female), age (0–15, 16–39, 40–64, 65–74, ≥75 years), number of calls from EMS teams to hospitals (once, twice, 3 times, 4 times, 5 times, ≥6 times), severity of illness (mild, moderate, severe, death), and destination hospital. The total EMS time was defined as the time from the EMS call to the time of arrival at the hospital and was divided into three categories: the response time (T1), on-scene time (T2), and transport time (T3). The definition of each category was as follows: T1 was the time from receipt of the EMS call to arrival of an EMS vehicle at the place where the patient was; T2 was the time from arrival of the EMS vehicle to departure from the patient's location; T3 was the time from departure from the patient's location to arrival at the hospital.

The emergency transport data used in this study are not available to the public because the Fire Department of Soma provided the data for the sole use of this research study due to the inclusion of personal information in the data.

### 2.3 Data analysis

The number of transport and the transition of each EMS time each month were illustrated in a line graph. The *t* test for continuous variables and x^2^ test for categorical variables were used to compare and analyze each characteristic of emergency transport between the previously evacuated area and the area outside the evacuation zone in Minamisoma City during the study period. Statistical analysis was performed using sex, age, time, severity, and the number of calls to the hospital as categorical variables and T1, T2, T3, and total EMS time as continuous variables. The categorical variables were analyzed using the chi-square test of Microsoft Excel (2016), and the continuous variables were analyzed using the *t* test. For T1, T2, T3, and total transport time in each region, histograms were used for confirmation. Two-sided tests of *P* values <.05 were considered statistically significant.

The primary outcome of this study was to compare the EMS transport time between the former evacuation zone and the areas outside the evacuation zone. The secondary outcome was to analyze each component of the EMS transport time and compare these between the 2 areas.

### 2.4 Ethics statement

This study was approved by the Minamisoma Municipal General Hospital Ethics Committee (approval number: 105) and the Fukushima Medical University Ethics Committee (reference number: General 30114). Individual consents of the participants in this study were waived by using the opt-out method.

## 3 Results

The characteristics of the patients are shown in Table 1. The number of emergency transports undertaken during the study period was 325 in the former evacuation zone and 4307 outside the evacuation zone. Of these, 197 (60.6%) and 2699 (62.7%) were elderly persons in the former evacuation zone and outside the evacuation zone, respectively. In the former evacuation zone, mild cases predominated (n = 163, 50.2%), whereas outside the evacuation zone, moderate cases predominated (n = 1977, 45.9%). There were 245 (75.4%) cases, which were accepted after one call from the EMS team to hospitals in the former evacuation zone and 3189 (74.1%) cases outside the evacuation zone.

**Table 1:** Characteristics of emergency transport in the former evacuation zone and outside the evacuation zone in Minamisoma City between July 12, 2016 and July 31, 2018.

	Former evacuation zone (n = 325)	Other areas (n = 4307)	*P*
Total EMS time, mean (SD), min	48 (16)	40 (15)	<.001
T1	8 (4)	9 (3)	.001
T2	23 (10)	21 (9)	.002
T3	16 (9)	9 (9)	<.001
Sex	0.345		
Male	183 (56.3%)	2310 (53.6%)	
Female	141 (43.4%)	1986 (46.1%)	
Age, y	0.546		
0–15	10 (3.1%)	173 (4.0%)	
16–39	32 (9.8%)	423 (9.8%)	
40–64	86 (26.5%)	1012 (23.5%)	
65–74	56 (17.2%)	685 (15.9%)	
≥75	141 (43.4%)	2014 (46.8%)	
Time of day			.655
00:00–05:59	37 (11.4%)	522 (12.1%)	
06:00–11:59	111 (34.2%)	1473 (34.2%)	
12:00–17:59	109 (33.5%)	1317 (30.6%)	
18:00–23:59	68 (20.9%)	995 (23.1%)	
Severity			.056
Mild (no need for hospitalization)	163 (50.2%)	1837 (42.7%)	
Moderate	132 (40.6%)	1977 (45.9%)	
Severe	24 (7.4%)	360 (8.3%)	
Death	6 (1.8%)	130 (3.0%)	
No. of calls to hospital			.809
Once	245 (75.4%)	3139 (74.1%)	
Twice	41 (12.6%)	622 (14.4%)	
3 Times	21 (6.5%)	254 (5.9%)	
4 Times	6 (1.8%)	117 (2.7%)	
5 Times	6 (1.8%)	59 (1.4%)	
≥6 Times	6 (1.8%)	61 (1.4%)	
Unknown	0	1	

SD = standard deviation.

**Figure 2:**
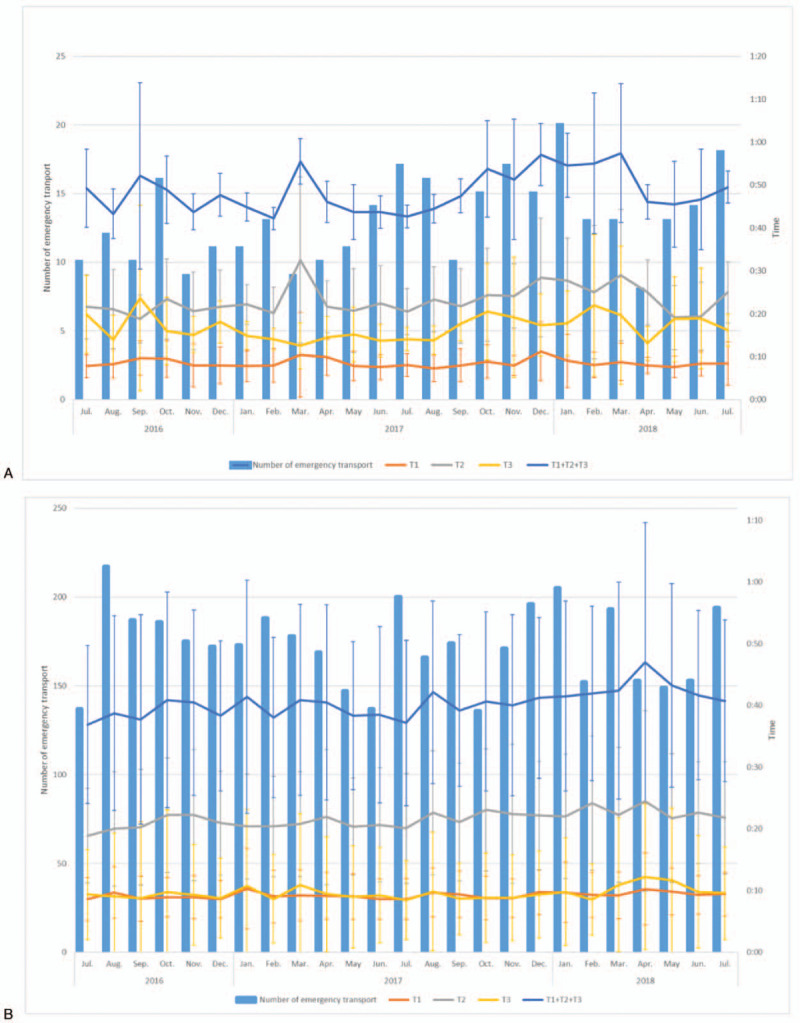
(A) Transition of each EMS time (mean and standard deviation) in the patients transferred from the former evacuation zone (n = 325). The average response time (T1), on-scene time (T2), transport time (T3), and total EMS time in the former evacuation zone in Minamisoma City are shown. During the study period, the number of EMS calls gradually increased, but no significant changes were detected for each EMS time. (B) Transition of each EMS time (mean and standard deviation" in the patients transferred from outside the evacuation zone (n = 4307). The average response time (T1), on-scene time (T2), transport time (T3), and total EMS time in the no-evacuation order zone in Minamisoma City are shown. During the study period, the number of EMS calls and each EMS time were generally stable.

Figure 2 shows the monthly demographics for the number of transports undertaken and the transition of each EMS time (mean and standard deviation) in the former evacuation zone and outside the evacuation zone. For T1 and T2, statistically significant changes were observed, which might not be clinically significant: former evacuation zone: T1, 8 ± 4 minutes, and T2, 23 ± 10 minutes; outside the evacuation zone: T1, 9 ± 3 minutes, and T2, 21 ± 9 minutes. The mean total EMS time and T3 time were significantly longer in the former evacuation zone than outside the evacuation zone: former evacuation zone: total, 48 ± 16 minutes, and T3, 16 ± 9 minutes; outside the evacuation zone: total, 40 ± 15 minutes, and T3, 9 ± 9 minutes; *P* < .001.

## 4 Discussion

In this study, we evaluated the characteristics of emergency transport services in the area where the evacuation orders were lifted after the FDNPP accident and compared emergency transport in the former evacuation zone and outside the evacuation zone. The mean transport time (T3) and total EMS response time were statistically significantly longer in the former evacuation zone. The total EMS time in the former evacuation zone was 8 and 9 minutes longer than outside the evacuation zone and the national average (39.3 minutes), respectively. Although the response time (T1) and on-scene time (T2) between the former evacuation zone and outside the evacuation zone were statistically significantly different, these differences were smaller than that of the transport time (T3) and the total EMS time, suggesting that they are not clinically significant.

The longer EMS times in the former evacuation zone could result from the smaller number of medical institutions available to accept the EMS transported patient in this area following their closure after the FDNPP accident. This finding is supported by a previous report on the effect of the FDNPP accident on EMS in the area.^[23]^ Since there is a lack of data on emergency transport before the FDNPP accident, the comparison of the EMS times before and after the disaster could not be assessed. However, the closure of medical institutions in the former evacuation zone and relocation of medical institutions from the evacuation zone to outside the evacuation zone may have impacted the longer EMS time in the former evacuation zone observed in this study. Additionally, geographical effects, such as an underpopulated area resulting from the disaster-affected areas of the FDNPP accident, may also have impacted the longer EMS time compared to the national average.

Appropriate distribution of EMS teams can mitigate the impact of nuclear disasters on emergency transport after lifting of evacuation orders. Although the total EMS time in the former evacuation zone was significantly longer, the differences between T1 and T2 in these 2 areas were smaller than that of T3. This finding may indicate that the time from the EMS call to the arrival at the patient's location and the departure from the patient's location were not greatly affected due to proper disposition of the EMS teams.

The mean total EMS time in the former evacuation zone (48 minutes) was also longer than the national average of 37.1 minutes. This is consistent with previous studies, which report that the closure of hospitals to accept EMS transported patients may have been one of the major reasons for the longer EMS times in areas where an evacuation order was lifted.^[23]^ Increasing the number of medical institutions may be the best way to address longer EMS times in the areas where evacuation orders are lifted. However, due to limited resources and low cost-benefit, this may not be possible. Other measures may focus on improving the communication between EMS teams and medical institutions to reduce the number of rejections of EMS reception and creation of a medical institution selection system before arrival at the patient's location to reduce the loss of EMS time. For example, the introduction of tools to facilitate smoother communication, such as smartphone applications, might be an option to solve this problem.^[31]^ Introduction of EMS vehicles with a doctor (eg, a doctor's car and doctor's helicopter) could also improve EMS systems instead of carrying patients to distant medical facilities.

The severity of cases transported by EMS was not significantly different between the two zones, with mild cases accounting for more than half (50.2%) of the total cases in the former evacuation zone. This suggests that residents may have requested an EMS even at lower disease severity. One plausible reason for this is that Odaka Hospital, which was the only hospital with an in-patient facility, had downscaled its function to offer only outpatient services. The majority of residents who returned after the reopening of Odaka Ward were the elderly; 49.5% of residents in the former evacuation zone of Odaka Ward were >65 years of age. Limited public transportation in Odaka Ward hindering movement of the elderly to the hospital may have increased the demand for EMS from elderly people with less severe diseases. To alleviate this point, it may be helpful to improve access to healthcare facilities among residents who live in the area where the evacuation order was lifted after long-term evacuation.

There was no significant difference in the age distribution of transferred patients between the two areas. However, given that the age distribution of the resident is actually different between Odaka Ward and other areas—the elderly were predominant in the returnees—younger people may have been transported from the former evacuation zone. This may be associated with the fact that after the evacuation order was lifted, the decontamination workers and other reconstruction workers were living in the former evacuation zone without being on the resident's card for that area.

This is the first study to compare and analyze emergency transport in the area where the evacuation order was lifted after the FDNPP accident. However, this study has some limitations. First, there was no data on emergency transport in this area before the FDNPP accident. Therefore, although the characteristics of emergency transport in this area before the accident may have been similar to those after the evacuation order was lifted, the characteristics before and after the accident could not be compared. Second, adjustment for baseline patient characteristics may be insufficient since only sex and age could be included in the analysis. Third, when comparing the results of this present study with the nationwide emergency transport, it is necessary to consider factors, such as the use of healthcare services, for example, medical expenses exemption, family composition, and availability of public transportation, which may differ from the rest of Japan precluding extrapolation of this study findings.

In conclusion, in areas where the evacuation orders were lifted after the FDNPP accident, the transport time from a patient's location to a hospital was significantly longer than that outside the evacuation zone, and subsequently, the total EMS time was also significantly longer in the former evacuation zone. Although the comparison of EMS time pre- and post-disaster could not be conducted due to the lack of data, this may be related to the decreased number of local medical institutions due to the closure of the facilities following the evacuation order after the nuclear accident. Given the low cost-benefits for establishing a new hospital and the limited human resources in the affected areas, it is important to develop locally adapted feasible countermeasures to address the limitations.

## Acknowledgments

The authors express our sincere gratitude to the Soma Regional Fire Headquarters and its staff. The authors are also grateful to Mr. Masatsugu Tanaki of Minamisoma Municipal General Hospital for his technical support and Mrs. Yuka Harada for her administrative support.

Additionally, the authors thank Editage (www.editage.com) for English language editing.

## Author contributions

Yamamoto C, Sawano T and Tsubokura M contributed to the conception and design of the research. Yamamoto C and Sawano T drafted the article. All authors performed critical revision of the article for intellectual content, were involved in interpretation of the cases, and approved submission of the article.

**Project administration:** Masaharu Tsubokura.

**Supervision:** Yoshitaka Nishikawa, Akihiko Ozaki, Yuki Shimada, Tomohiro Morita, Arifumi Hasegawa, Tomoyoshi Oikawa, Masaharu Tsubokura.

**Writing – original draft:** Chika Yamamoto, Toyoaki Sawano.

**Writing – review & editing:** Tianchen Zhao, Masaharu Tsubokura.

## Supplementary Material

Supplemental Digital Content
