# Supplementary material for: Evaluation of the emergency medical system in an area following lifting of the mandatory evacuation order after the Fukushima Daiichi Nuclear Power Plant accident: A retrospective cross-sectional observational study
Source: Medicine (Baltimore). 2021 Jun 25;100(25):e26466. doi: 10.1097/MD.0000000000026466 (PMC8238363; doi:10.1097/MD.0000000000026466)
Supplement: Supplemental Digital Content [file medi-100-e26466-s001.docx]

Supplemental material

**List of all of the variables in the emergency transport record from the Fire Department of Soma**

Since there were many variables in the emergency data that the Fire Department of Soma provided, we have listed all of the variables in this supplemental material as follows.

Year

Month

Case number

Station number

Name of the station

Name of ambulance team

Transport category name

Reason for non-transportation

Name of accident type

Location of the accident

Name of destination

Name of express-way

Name of awareness method

Weather

Time of emergency call

Time of command

Time of departure

Time of arrival at the scene

Time of contact with injured/ill person

Time of admission of injured/ill person to an emergency vehicle

Time of departure from the scene

Time of arrival at a hospital

Time of admission of injured/ill person to a hospital

Time of withdrawal from a hospital

Time of return

National Holidays or not

Name of the day of the week

Month

Day

Time of day

Name of time period

Organization requesting hospital transfer

Reason for hospital transfer

Use of doctor car /helicopter operations

Classification of doctor car/helicopter operation

Name of the operation

Name of vehicle

Name of ambulance commander

Name of engineer

Name of team members

Name of emergency medical technician (EMT) on board

Name of physician on scene

Condition of the injured person -foreign body in the airway

-unconscious

- immobile

-chest pain,

-dyspnea

-external bleeding

-others

Verbal instructions -continued observation

-positioning

-securing airway

-removal of foreign body

-artificial respiration

-cardiac massage

-defibrillation

-hemostasis of external bleeding

-head immobilization

-immobilization of fracture

-cleaning and dressing of wound

movement

-others

Departure - free description

Outline of the accident

Main injured/ill person

Injured/ill person number

Injured/ill person - accident type name

- name of transport category

- name of reason for non-transport

Age group name

Age

Name of gender

Name of place of residence

Place

Floor level of classification

Floor level

Time of determination of destination hospital

Number of contacts until decision

Transport organization-name

name by notification

name by establishment

name by jurisdiction

name by location

Name of department

Duty hospital or not

Date of arrival at hospital

Level of emergency by EMT

Level of injury/illness

Name of disease classification

Confirmed diagnosis

Name of injury or disease

Situation at the time of contact

Outline of the accident and the main complaint/situation, etc.

Emergency procedures-hemostasis

-immobilization

-artificial respiration

-cardiac massage

-cardiac massage (automatic)

-cardiopulmonary resuscitation

-cardiopulmonary resuscitation (automatic)

-oxygen inhalation

-airway securing (nasal airway, foreign body removal using instruments, LM, etc., airway intubation)

- heat retention

-dressing

-home therapy (intravenous drip, external fistula, etc.)

First aid -continued observation

-position control

-securing airway

-removal of foreign body

-artificial respiration

-cardiac massage

-defibrillation

-hemostasis of external bleeding

-head immobilization

-immobilization of fracture

-wound cleaning and dressing,

-transfer

-others

Number of transfers

Specific action -defibrillation

-airway securing

venous route securing

-drug administration

Presumed cause of cardiopulmonary arrest

Restarted heartbeat

One-month survival

Brain function category

All functional categories

Corpse signs
